# Impact of Bariatric Surgery on Unplanned Hospital Admissions for Infection

**DOI:** 10.1007/s11695-022-05975-4

**Published:** 2022-04-04

**Authors:** Tim Cundy, Greg D. Gamble, Elaine Yi, Nicholas Evennett, Grant Beban

**Affiliations:** 1 Bariatric Surgical Service, Auckland City Hospital, Auckland, New Zealand; 2 Department of Medicine, Faculty of Medical & Health Sciences, University of Auckland, Private Bag 92019, Auckland, New Zealand

**Keywords:** Bariatric surgery, Hospital admissions, Skin and soft tissue infection, Upper respiratory, Urinary tract infection

## Abstract

**Purpose:**

Both obesity and type 2 diabetes are associated with an increased risk of skin and soft tissue (SSTI), urinary tract, and lower respiratory tract infections but it is not clear whether the incidence of such infections is reduced after bariatric surgery.

**Materials and Methods:**

In people accepted onto our publicly funded bariatric program, we recorded unplanned admissions to public hospitals over a median follow-up of 4.5 years in those successfully undergoing surgery and in those who withdrew from the program. Rates of admission for the composite outcome (SSTI, urinary tract, or lower respiratory infection) were compared.

**Results:**

Of 774 people accepted onto the program, 49% underwent surgery. Infections accounted for 27% of unplanned admissions in those not completing surgery and 13% of those who underwent surgery (*p* < 0.001). The rate of admission was 60% lower in people who underwent surgery than those who did not: 4.3 vs 12.2 per 100 patient-years (*P* < 0.002), a difference maintained across 8 years’ follow-up. The impact of surgery was independent of enrolment age, BMI, or diabetes and smoking status. Of the three types of infection in the composite outcome, SSTI were the most prevalent and showed the greatest reduction (*p* < 0.0001). The median day stay for infection was 0.5 day less in those who underwent surgery (*p* < 0.01).

**Conclusions:**

Hospitalization for these three infectious diseases in people undergoing bariatric surgery was lower than that in people enrolled in the bariatric program but not completing surgery. The effect was greatest for SSTI, and sustained to at least 8 years.

**Graphical abstract:**

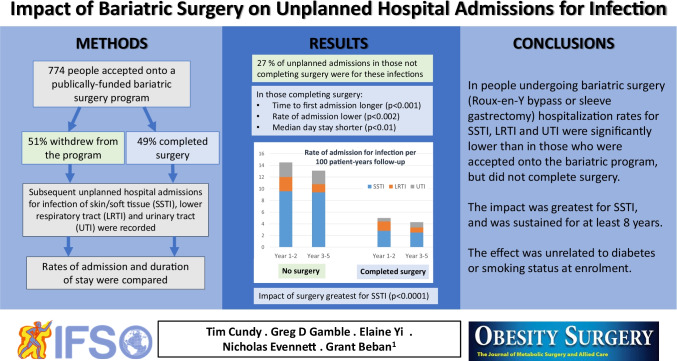

**Supplementary Information:**

The online version contains supplementary material available at 10.1007/s11695-022-05975-4.

## Introduction

Bariatric surgery is the most effective treatment for the maintenance of weight loss in people with severe obesity. After bariatric surgery, rapid improvements in cardiovascular risk factors such as dyslipidemia, hypertension, and glucose intolerance/type 2 diabetes are seen, and in the long term, this can translate into reduced risk for primary and secondary cardiovascular events and cardiovascular deaths [1–3]. A majority of people with obstructive sleep apnea also benefit from surgery [4], as do those with non-alcoholic fatty liver disease [5], and the incidence of end stage renal disease may also be reduced [6]. There may be other obesity-related conditions that could also benefit from weight loss.

Both obesity and type 2 diabetes are associated with an increased risk of infections of the skin and soft tissue, urinary tract, and lower respiratory tract [7–15] but there is relatively little published information on whether the incidence of such infections is reduced after bariatric surgery, and if so how long the effect is maintained. We hypothesized that bariatric surgery would be associated with a lower rate of unplanned hospital attendances for medical conditions, particularly those due to skin or soft tissue infection (SSTI), urinary tract infection (UTI), and lower respiratory tract (LRTI). As we have reported previously, approximately half of those accepted for surgery in our publicly-funded bariatric program for various reasons do not proceed with surgery [16]. In this study, we have used data from the latter group for comparison with those that did undergo surgery.

## Material and Methods

The publicly-funded bariatric program at Auckland City Hospital was first established in 2006. Prospective candidates for surgery were referred to the bariatric service by either an independent general practitioner or specialist. Eligibility was based on their medical profile and the likelihood of optimum surgery results. The criteria for acceptance included a BMI ≥ 35 kg/m^2^, a history of failed weight-loss attempts, and at least one obesity-related comorbidity (most commonly type 2 diabetes or sleep apnea). On acceptance onto the program, candidates attended appointments with a surgeon, a health psychologist, a dietitian, and a nurse specialist and had regular follow-up with team members until surgery, which on average was about 1 year after referral.

In this prospective cohort study, we examined hospital attendances of all subjects who had been referred to the bariatric program and had been accepted as surgical candidates from the start of the program through to December 31st, 2016. In this period, 774 people were accepted onto the bariatric program of whom 378 (49%) successfully underwent surgery and 396 withdrew from the program and did not proceed with surgery. Only two bariatric procedures were undertaken: either sleeve gastrectomy (53%) or Roux-en-Y gastric bypass (47%).

Information on attendances at all public hospitals in the northern region of New Zealand was gathered from the shared medical record. Information on hospital attendances at private facilities or hospitals outside the northern region was not available. Outcomes in the two groups—those who underwent surgery and those who did not—were compared.

### Data Collection

The shared medical record included data from the TestSafe system that archives all community medical laboratory tests and the lodging of prescriptions at community pharmacies. Follow-up started from the day of the procedure in those completing surgery, or from the day of referral in those not completing surgery and was continued to 31st December 2018 or the last day of contact prior to that date. The last day of contact was defined as whichever was the most recent of either a hospital communication indicating that the patient had been seen, a laboratory test completed, or a prescription lodged with a pharmacy.

Each hospital attendance was reviewed and classified as either planned (elective) or unplanned (non-elective), and the duration of admission was recorded. Discharge summaries were scrutinized and the major reason for admission was classified according to Supplementary Table 1. We collected annual data on weight and calculated body mass index (BMI; kg/m^2^).

### Endpoints

The main outcome was unplanned hospital attendance with SSTI, UTI, or LRTI as a composite outcome. Post-operative infections of surgical sites were counted as complications of bariatric surgery and not included in the composite outcome. We looked at several parameters: the time to first admission with one of these infections; the number of patients with any admission for these infections; the number of infection admissions/100 patient-years of follow-up and the median length of stay in hospital.

### Statistical Analysis

Between group comparisons were sought using Student’s *t*-test, *Χ*^2^/Fisher’s exact tests, or Wilcoxon test as appropriate for the distributions. Between group differences are presented as mean (95% confidence interval) or median difference (95% confidence interval from Hodges-Lehmann estimate). A mixed model approach to repeated measures was used to examine the change in body mass index over time. Baseline BMI was included as a covariate and a covariance structure was chosen from the model with the lowest Akaike information criterion fitted from unstructured, compound symmetry or first-order autoregressive. Significant main or interaction effects were further explored using the method of Tukey. All-cause mortality was compared between groups using the Kaplan–Meier approach. These analyses were performed using the procedures of SAS (v9.4 SAS Institute Inc., Cary, NC, USA). Rate, rate ratios, and differences per 100 patient years with their 95% confidence intervals (Mid-P exact method) were calculated using Openepi. All tests were two tailed and *P* < 0.05 was considered significant.

### Ethical Considerations

The study was approved by the New Zealand Health & Disability Ethics Committee (18-NTA186) and the Auckland District Health Board Research office.

## Results

### Demography

Forty-nine percent of people accepted onto the program underwent surgery and 51% withdrew from the program. The baseline characteristics of the two groups (undergoing or not completing surgery) are shown in Table 1. At referral, the two groups were similar in mean age and in the proportion with obstructive sleep apnea or diabetes. Of those with diabetes, the great majority (99.4%) had type 2 diabetes.

**Table 1:** Baseline data comparing subjects completing surgery with those that did not

	Completed surgery	Did not complete surgery	*P* values
Number	378	396	
Female, *n* (%)	262 (70%)	217 (55%)	***P* < 0.0001**
Age	44.3 (9.5)	42.8 (10.0)	0.067
Ethnicity			
- European - Māori - Other - Pasifika	180 (48%) 81 (21%) 46 (12%) 71 (19%)	110 (28%) 71 (18%) 47 (12%) 168 (42%)	***P* < 0.0001**
Body mass index (kg/m^2^)	47.0 (6.8)	49.1 (9.2)	**0.0061**
Smokers	43 (11.5%)	71 (18.1%)	**0.011**
No. with diabetes	227 (60%)	239 (60%)	0.9
No. on insulin treatment	73 (32%)	71 (30%)	0.61
HbA1c at referral – mmol/mol Non-diabetic (n = 308)	39 (5)	41 (5)	**0.003**
HbA1c at referral – mmol/mol Diabetic (*n* = 466)	66 (18)	70 (21)	**0.02**
No. with obstructive sleep apnea	126 (34%)	140 (37%)	0.32
No. without OSA or diabetes	78 (21%)	91 (23%)	0.44
Surgical procedure			
Sleeve gastrectomy	200 (53%)	-	
Roux-en-Y gastric bypass	178 (47%)	-	
Duration of follow-up (months)	55 (34) *52 (0.5, 130)	62 (32) *56 (2.0, 129)	**0.0003**
Died during follow-up	1 (0.3%)	14 (3.5%)	**0.0009**

Data given as mean (SD) or *median (min, max)

*OSA* obstructive sleep apnea

As we have previously reported, men were less likely to undergo surgery than women, and those withdrawing from the program had a greater mean BMI, were on average younger, were more likely to be of Pasifika (Pacific Island) origin, and had higher mean glycated hemoglobin (HbA1c) [16]. In those not proceeding with surgery, the median duration of follow-up was slightly longer, because the timing at which follow-up began differed between the groups (time of acceptance onto program vs time of surgery).

### Change in BMI and Survival

**Fig. 1:**
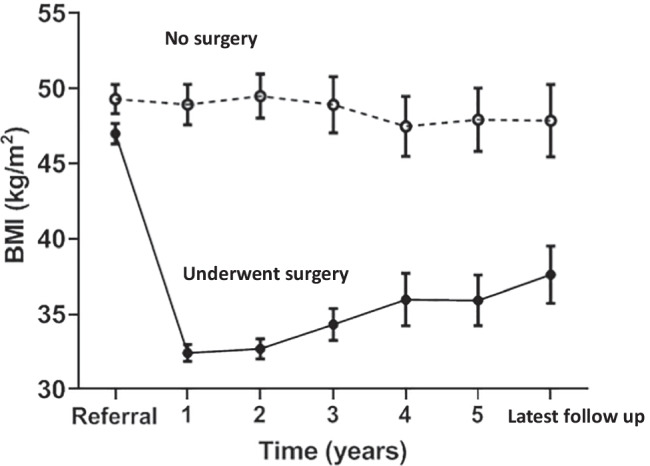
Sequential change in body mass index (BMI) in the those undergoing bariatric surgery (solid line) and those who did not complete surgery (dotted line). Data are given as mean with standard deviation

The mean BMI remained unchanged over follow-up in those who withdrew from the program. In those completing surgery, the mean BMI was 15 kg/m^2^ lower at 1 year and thereafter gradually increased, but by 5 years still remained a mean 10 kg/m^2^ lower than before surgery (Fig. 1). The change in BMI did not differ according to the type of surgery undertaken. Survival was better in those who underwent surgery than in those withdrawing from the program (*p* = 0.0004).

### Admissions with Infection

Over a follow-up of up to 8 years (median 4.5), 54 (14%) people who had surgery and 123 (31%) who did not have surgery had unplanned admissions to hospital for SSTI, UTI, or LRTI (*P* < 0.001). There were 79 admissions for infection in people who had surgery and 224 in people who did not have surgery. The time to first admission was significantly shorter in those who did not have surgery (*P* < 0.001), and the rate of admission was 60% lower in people who had surgery than those who did not: 4.3 vs 12.2 admissions per 100 patient-years (*P* < 0.002)—a difference that was maintained across 8 years’ follow-up (Table 2). Of the three types of infection in the composite outcome, SSTI were the most prevalent. Although there were reductions in rates of admission across all three type of infection, the number of admissions for SSTI showed the greatest decrement (*p* < 0.0001) with smaller effects on LRTI (*p* = 0.041) and UTI (*p* = 0.47) (Table 2).

**Table 2:** Rates of admission during follow-up with different types of infection according to whether or not bariatric surgery was completed

	Admissions with infection/100 patient-years		
	Year 1–2	Year 3–5	Year 6–8
Bariatric surgery – not completed			
Patient-years	760	812	376
Skin/soft tissue	73 (**9.6**; 7.6, 12.0)	76 (**9.4**; 7.4, 11.7)	26 (**6.9**; 4.6, 10.0)
Urinary tract	18 (**2.4**; 1.5, 3.7)	11 (**1.4**; 0.7 2.4)	0 -
Lower respiratory	19 (**2.5**; 1.6, 3.8)	19 (**2.3**; 1.5, 3.6)	10 (**2.7**; 1.4, 4.7)
All infections	110 (**14.5**; 12.0, 17.4)	106 (**13.1**; 10.7 15.7)	36 (**9.6**; 6.8, 13.1)
Bariatric surgery – completed			
Patient-years	639	645	322
Skin/soft tissue	18 (**2.8**; 1.7, 4.4)	16 (**2.5**; 1.5, 3.9)	8 (**2.5**; 1.2, 4.7)
Urinary tract	10 (**1.6**; 0.8, 2.8)	6 (**0.9**; 0.4, 1.9)	5 (**1.6**; 0.6, 3.4)
Lower respiratory	4 (**0.6**; 0.2, 1.5)	6 (**0.9**; 0.4, 1.9)	5 (**1.6**; 0.6, 3.4)
All infections	32 (**5.0**; 3.5, 7.0)	28 (**4.3**; 2.9, 6.2)	15 (**4.7**, 2.7, 7.5)

Data given are the number of admissions for infection and the rate of admissions/100 patient years (bold); with 95% CI

The proportion of each group that had any admission for SSTI, UTI, or LRTI was also reduced significantly: 3.4% of those completing surgery vs 8.8% of those not completing surgery (*p* = 0.0007). The proportion of non-elective admissions attributed to SSTI, UTI, or LRTI was significantly lower in those completing surgery than in those not completing surgery (12.8 vs 26.9%, *P* = 0.0008).

The apparent impact of surgery on rates of admission for infection was the same (P_(interaction)_ = 0.78) irrespective of diabetes status (Table 2), sex, above median BMI, or above median age at enrollment, or smoking status (Supplementary Table 2, all P_(interaction)_ > 0.54).

The median length of in-patient stay was 2 days (IQR 1–4) in those who underwent surgery and 3 days (IQR 2–6) in those not having surgery (*p* < 0.007). The Hodges-Lehmann estimate for the median difference in stay was − 0.5 day (95% CI 0 − 1).

## Discussion

In this study, we found that people who underwent bariatric surgery had significantly fewer admissions and slightly shorter day stay than people accepted onto the bariatric program who did not complete surgery. The three categories of infectious disease we studied accounted for 1 in 4 unplanned admissions in those not completing surgery but 1 in 8 in those completing surgery. The apparent impact of surgery was sustained for several years. The greatest impact was on the most prevalent of these infections—skin and soft tissue infection (cellulitis)—but there were also reductions rates of admission for UTI and LRTI, though the study was probably underpowered to demonstrate statistical significance. As discussed previously [16], the control group (those accepted onto the program but not completing surgery) differed in some measures from the surgery group. In particular, the former had a higher mean BMI and had a higher proportion of men and were slightly younger and more likely to be smokers. However, analysis by gender, BMI category, age, and smoking status did not attenuate the apparent beneficial effect on hospitalization with infection.

Both diabetes and obesity are risk factors for all three infectious diseases included in the composite outcome. People with type 2 diabetes tend to have more frequent admissions (and re-admissions) and longer lengths of stay in hospital [10, 12, 13]. As expected, surgery was associated with a high rate of remission of diabetes. In this cohort, 80% had early remission of diabetes after surgery, and 5 years after surgery, 60% remained in remission [17], but this alone cannot explain the lower rates of infection, as the apparent impact of surgery was also seen in those without diabetes at the time of acceptance onto the program (Table 3).

**Table 3:** Rates of admission with infection during follow-up according to diabetes status at acceptance onto the program and whether or not bariatric surgery was completed

Category		Admissions with infection/100 patient-years		
Diabetes	Surgery	Year 1–2	Year 3–5	Year 6–8
No	No	42 [297] (**14.1**; 10.3, 18.9)	40 [316] (**12.7**; 9.2,17.1)	11 [147] (**7.5**; 3.9, 13.0)
No	Yes	9 [283] (**3.2**; 1.6, 5.8)	14 [294] (**4.8**; 2.7, 7.8)	5 [156] (**3.2**; 1.2, 7.1)
Yes	No	67 [461] (**14.5**; 11.4, 18.4)	66 [494] (**13.3**; 10.4, 16.9)	25 [229] (**10.9**; 7.2, 15.9)
Yes	Yes	23 [410] (**5.6**; 3.6, 8.3)	14 [352] (**4.0**; 2.3, 6.5)	10 [167] (**6.0**; 3.1, 10.7)

Data are the number of admissions for infection; number of patient-years of follow-up (square brackets) and the rate of admissions/100 patient years (bold); with 95% CI

There have been relatively few similar studies. Zingmond et al. [18] and Goto et al. [19] comparing the same population before and after surgery found reduced rates of hospitalization for SSTI and LRTI over 2–3 years’ follow-up, but in the latter paper but the rate of admission for UTI was increased, compared to preoperative rates. Han et al. [20] reported a lower risk of UTI in people with heart failure who had undergone bariatric surgery than those who did not. Sharma et al. [21] reported a lower risk of LRTI in people with celiac disease who had undergone bariatric surgery than those who did not. Chen et al. [22] also reported a reduced risk of hospitalization with LRTI, and a shorter length of stay compared to a propensity-matched control group who did not have surgery. In our study, we were able to determine that the impact of surgery on hospitalization for these infections was independent of pre-surgery BMI and diabetes and smoking status, and was sustained to at least 5 to 8 years (Table 3, Supp Table 2).

Possible mechanisms include, in the case of diabetes, an increased susceptibility to infection through impairment of neutrophil functioning and antioxidant systems. The biological mechanisms of the increased risk of infection among those with high BMI remains poorly understood. Again, there may be a variety of immune mediators, but in the case of SSTI in particular, mechanical factors resulting in impaired venous and lymphatic drainage and impaired skin barrier repair are likely to be important.

There are limitations to the study. It was relatively small in size, and we do not have data on rates of admission for infection before enrolment in the bariatric program. It was not a randomized trial, because the control group which comprised people dropping out of the program differed in some important respects from the operated group. The attrition rate in our cohort with 51% not completing surgery is not atypical [23]. Reasons why candidates may not complete bariatric surgery include cost, insurance coverage, travel distances, surgical wait time, fear of risks, concerns about adapting to eating post-operatively, and the need to take time off work [16,23]. In our cohort, those dropping out of the program were more likely to be men and to be smokers, and had a lower mean age and higher BMI [16]. We examined the impact of factors that differed between the groups (gender, age, diabetes and smoking status, and pre-operative BMI) and conclude that these differences could not explain the impact of bariatric surgery on hospital admissions for infection.

## Conclusions

We found that hospitalization for these three infectious diseases in people undergoing bariatric surgery was lower than that in people enrolled in the bariatric program but not completing surgery. The effect was greatest for SSTI, and was sustained long term. This represents a significant health and cost benefit of surgery in those with frequent infections, particularly SSTI.

## Supplementary Information

Below is the link to the electronic supplementary material.

Supplementary file1 (DOCX 35 kb)
