# Supplementary material for: Impact of Bariatric Surgery on Unplanned Hospital Admissions for Infection
Source: Obes Surg. 2022 Apr 4;32(6):1896–901. doi: 10.1007/s11695-022-05975-4 (PMC9072262; doi:10.1007/s11695-022-05975-4)
Supplement: Supplementary file 1 — Supplementary file1 (DOCX 35 kb) [file 11695_2022_5975_MOESM1_ESM.docx]

**Supplementary** **Table 1**

Categories for primary reason for hospital admission

| **Primary reason for admission** | Coding |
| --- | --- |
| **Medical** |  |
| Cardiac – heart failure | 11 |
| Cardiac – ischaemic | 12 |
| Cardiac - other | 13 |
| Stroke/TIA | 14 |
| Renal | 15 |
| Diabetes | 16 |
| Diabetes foot | 17 |
| Infectious disease respiratory | 18 |
| Other respiratory | 19 |
| Infectious disease soft tissue | 20 |
| Infectious disease urinary/renal | 21 |
| Other medical | 22 |
|  |  |
| **Surgical** |  |
| Bariatric-related | 31 |
| Other gastrointestinal | 32 |
| Orthopaedic | 33 |
| Ophthalmic | 34 |
| Transplant | 35 |
| Gynaecological | 36 |
| Obstetric | 37 |
| Other surgery | 38 |
|  |  |
| **Other specialties** |  |
| Psychiatric | 51 |
| Other | 52 |

The composite outcome of admission with infection included

categories 18, 20 and 21.

**Supplementary Table 2.** Rates of admission with infection during follow-up according to gender, median BMI and age at acceptance onto the program, gender, and smoking status according to whether or not bariatric surgery was completed.

| **Category** | | **Admissions with infection/100 patient-years** | | |
| --- | --- | --- | --- | --- |
| **Gender** | **Surgery** | **Year 1-2** | **Year 3-5** | **Year 6-8** |
| Male | No | 51 [345]  (**14.8**; 11.1, 19.3) | 58 [376]  (**15.4**; 11.8, 19.8) | 16 [161]  (**9.9**; 5.9, 15.8) |
| Male | Yes | 11 [485]  (**2.3**; 1.2, 3.9) | 5 [460]  (**1.1**; 0.4, 2.4) | 3 [230]  (**1.3**; 0.3, 3.6) |
| Female | No | 59 [415]  (**14.2**; 10.9, 18.2) | 49 [436]  (**11.2**; 8.4, 14.7) | 20 [215]  (**9.3**; 5.8, 14.1) |
| Female | Yes | 21 [485]  (**4.3**; 2.8, 6.5) | 23 [460]  (**5.0**; 3.2, 7.4) | 12 [230]  (**5.2**; 2.8, 8.9) |

| **BMI (kg/m^2^)** | **Surgery** | **Year 1-2** | **Year 3-5** | **Year 6-8** |
| --- | --- | --- | --- | --- |
| < 47.2 | No | 39 [330]  (**11.8**; 8.5, 16.0) | 52 [363]  (**14.3**; 10.8,18.6) | 20 [180]  (**11.1**; 7.0, 16.9) |
| < 47.2 | Yes | 22 [367]  (**6.0;** 3.9, 8.9) | 15 [340]  (**4.4**; 2.6, 7.1) | 7 [186]  (**3.8**; 1.6, 7.4) |
| > 47.2 | No | 67 [385]  (**17.4**; 13.5, 22.0) | 50 [396]  (**12.6**; 9.5, 16.5) | 16 [162]  (**9.9;** 5.8, 15.7) |
| > 47.2 | Yes | 10 [323]  (**3.1**; 1.6, 5.5) | 13 [305]  (**4.3**; 2.4, 7.1) | 8 [136]  (**5.9**; 2.7, 11.2) |

| **Age (yr)** | **Surgery** | **Year 1-2** | **Year 3-5** | **Year 6-8** |
| --- | --- | --- | --- | --- |
| < 44.4 | No | 49 [386]  (**12.7**; 9.4, 16.6) | 46 [405]  (**11.4;** 8.4, 15.0) | 15 [180]  (**8.3**; 4.8, 13.4) |
| < 44.4 | Yes | 17 [329]  (**5.2**; 3.1, 8.1) | 15 [322]  (**4.7**; 2.7, 7.5) | 8 [167]  (**4.8**; 2.2, 9.1) |
| > 44.4 | No | 61 [372]  (**16.4**; 12.7, 20.9) | 60 [404]  (**14.9**; 11.4, 19.0) | 21 [193]  (**10.9**; 6.9, 16.4) |
| > 44.4 | Yes | 15 [363]  (**4.1**; 2.4, 6.7) | 13 [320]  (**4.1**; 2.3, 6.8) | 7 [153]  (**4.6**; 2.0, 9.1) |

| **Smoking** | **Surgery** | **Year 1-2** | **Year 3-5** | **Year 6-8** |
| --- | --- | --- | --- | --- |
| No | No | 93 [614]  (**15.2**; 12.3, 18.5) | 74 [645]  (**11.5**; 9.1, 14.3) | 24 [289.0]  (**8.3**; 5.4, 12.2) |
| No | Yes | 28 [610]  (**4.6**; 3.1, 6.5) | 20 [559]  (**3.6**; 2.2, 5.4) | 11 [290.0]  (**3.8**; 2.0, 6.6) |
| Yes | No | 17 [141]  (**12.1**; 7.2, 1.9) | 32 [162]  (**19.8**; 13.7, 27.6) | 12 [84.1]  (**14.3**; 7.7, 2.4) |
| Yes | Yes | 4 [76]  (**5.3**; 1.7, 12.7) | 8 [81]  (**9.9**; 4.6, 18.7) | 3 [30.4]  (**9.9**; 2.5, 26.9) |

Data are the number of admissions for infection; number of patient-years of follow up [square brackets] and the rate of admissions/100 patient years (bold); with 95% confidence intervals.
